# CUSTOMIZING STAKEHOLDER ENGAGEMENT TO SUPPORT AGING IN PLACE

**DOI:** 10.1093/geroni/igz038.2975

**Published:** 2019-11-08

**Authors:** Elyse Perweiler, Jennifer DeGennaro, Sherry Pomerantz, Lisa Bodenheimer, Marilyn Mock, Margaret Avallone

**Affiliations:** 1 Rowan School of Osteopathic Medicine, Stratford, New Jersey, United States; 2 Rowan University School of Osteopathic Medicine, Stratford, New Jersey, United States; 3 Fair Share Housing Inc., Northgate II, Camden, New Jersey, United States; 4 Rutgers University School of Nursing-Camden, Camden, New Jersey, United States

## Abstract

Geriatrics infusion and transformation of community-based settings to support “aging in place” is complex. It requires a customized approach that engages multiple stakeholders who are invested in systems redesign and process change. Rowan University School of Osteopathic Medicine’s NJ Geriatrics Workforce Enhancement Program, in partnership with an affordable housing facility operated by Fair Share Housing/Northgate II, and Rutgers University School of Nursing (RSoN), implemented a Resident Health Risk Assessment (RHRA) tool as part of an interprofessional community-based training experience for health professions students. The goal was to identify health risks that impact a resident’s ability to age in place and implement a person-centered intervention plan. Multi-level stakeholder engagement and ongoing rapid cycle quality improvement catalyzed changes in structure and process for all partners. Results included refinement of an interprofessional clinical rotation, introduction of competency attainment into the orientation process, and resource reallocation to support data collection.

